# Health relevance of lowering postprandial glycaemia in the paediatric population through diet’: results from a multistakeholder workshop

**DOI:** 10.1007/s00394-022-03047-y

**Published:** 2022-12-19

**Authors:** Sophie Vinoy, Janina Goletzke, Maryam Rakhshandehroo, Lisa Schweitzer, Matthieu Flourakis, Antje Körner, Ute Alexy, Evert M. van Schothorst, Antonio Ceriello, Julia K. Zakrzewski-Fruer, Anette Buyken

**Affiliations:** 1 Mondelēz International, Nutrition Research, Clamart, France; 2 Paderborn University, Paderborn, Germany; 3 Danone Nutricia Research, Utrecht, The Netherlands; 4 BENEO GmbH, Mannheim, Germany; 5 ILSI Europe, Brussels, Belgium; 6 Leipzig University, Leipzig, Germany; 7 University of Bonn, Bonn, Germany; 8 Human and Animal Physiology, Wageningen University, Wageningen, The Netherlands; 9 IRCCS Multimedica of Milan, Milan, Italy; 10 University of Bedfordshire, Luton, UK

**Keywords:** Glycaemic response, Infant, Children, Adolescent, Workshop, Glycaemic index, Glycaemic load

## Abstract

To summarize current knowledge and gaps regarding the role of postprandial glycaemic response in the paediatric population, a workshop was organized in June 2021 by the European branch of the International Life Science Institute (ILSI). This virtual event comprised of talks given by experts followed by in-depth discussions in breakout sessions with workshop participants. The main pre-specified topics addressed by the workshop organizing committee to the invited speakers and the workshop participants were: (1) the role of glycaemic responses for paediatric health, based on mechanistic insights from animal and human data, and long-term evidence from observational and intervention studies in paediatric populations, and (2) changes in metabolism and changes in dietary needs from infancy to adolescence. Each talk as well as the discussions were summarised, including the main identified research gaps. The workshop led to the consensus on the crucial role on health of postprandial glycaemic response in paediatric population. However, a lack of scientific data has been identified regarding detailed glucose and insulin profiles in response to foods commonly consumed by paediatric populations, as well as a lack of long-term evidence including the need for suitable predictors during childhood and adolescence to anticipate health effects during adulthood.

**Supplementary Information:**

The online version contains supplementary material available at 10.1007/s00394-022-03047-y.

## Introduction

The glycaemic index (GI) ranks carbohydrate-rich foods by their capacity to elevate blood glucose levels during postprandial states for a fixed amount of available carbohydrates [1, 2]. The GI is, therefore, both a standardised glycaemic response (GR) (based on an equal amount of available carbohydrates), and a relative GR compared to a reference food [3]. Diets that produce smaller postprandial GR and insulinaemic responses (IR) are associated with a wide range of health benefits, including improved insulin secretion and sensitivity, and thus enhanced glycaemic control, in adults [4–6]. Specifically, diets with a high GI and/or glycaemic load (GL: the overall glycaemic impact of the carbohydrates in a diet) are associated with an increased risk of type 2 diabetes (T2D) [7] and cardiovascular events [8, 9] in adulthood. For both associations, substantial proof using robust statistical models underpins their causality [7, 10]. In addition, the European Food Safety Authority considers the reduction of postprandial GR physiologically positive for health, if it is not associated by a disproportionate increase in insulin [11]. In children with obesity, lowering postprandial GR, by eating a low GI diet showed some beneficial effects on lipid profile and BMI Z-score [12]. Yet, a clear consensus on the potential short- and longer-term benefits of lowering GR in paediatric populations (i.e., infants, children and adolescents) is currently lacking [13], i.e., the major pieces of scientific evidence have been established in adult populations. The complex interaction of glycaemia with other responses, e.g., elevated inflammatory markers and adipokine concentrations, especially among children and adolescents, is potentially important in understanding and preventing later metabolic disease during adulthood [14]. Furthermore, the paediatric period is characterised by additional complexity due to evolving metabolic regulations during infancy, childhood, and adolescence, which may entail specific needs regarding dietary intake [15].

The primary aim of this workshop was to share consolidated scientific knowledge and recent advances from mechanistic studies as well as intervention and cohort studies in paediatric populations examining the relevance of limiting high glycaemic excursions for health outcomes, with a specific focus on glycaemic control and cardiometabolic risk markers. The secondary aim was to identify gaps and challenges associated with research in these age groups (infants, children and adolescents) and to discuss which kind of studies are needed to meet these challenges.

## Methods: design of the workshop and extraction of themes

**Fig. 1:**
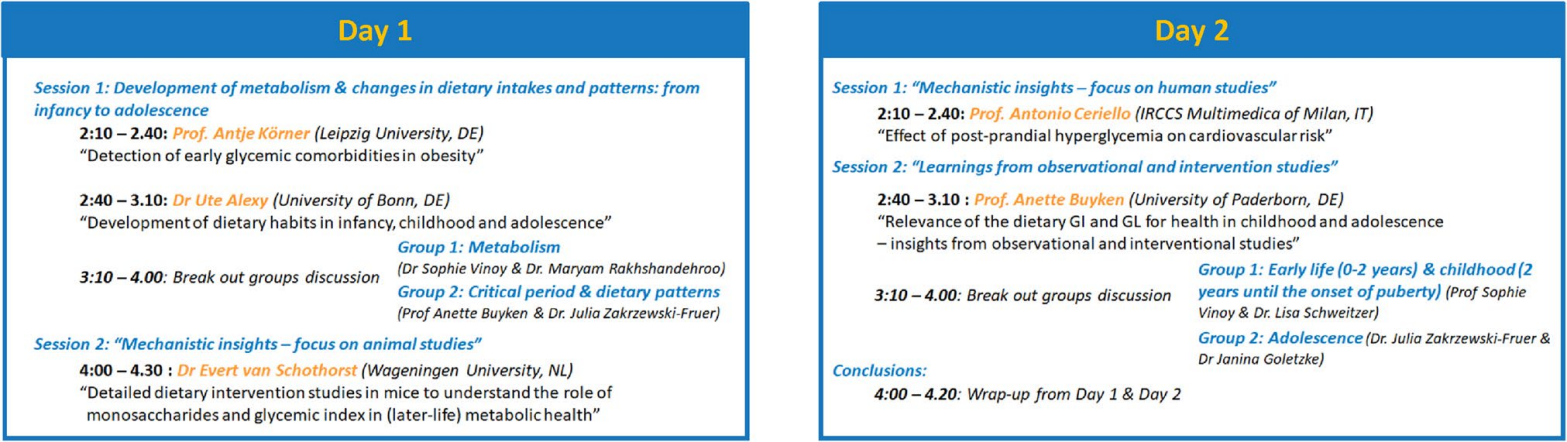
Programme of the 2 day workshop

This workshop was organized by the European branch of the International Life Science Institute (ILSI) in a virtual delivery mode on two consecutive days, the 30th of June and 1st of July 2021. Representatives from academia and industry were invited to participate. The workshop consisted of talks given by experts followed by in-depth discussions in breakout sessions. The full workshop programme is shown in Fig. 1. The three key sessions addressed:

1. Mechanistic insights to understand the role of glycaemic response in metabolic health in paediatric populations using animal and human data.
2. Learnings from observational and intervention studies on the impact of dietary GI and glycaemic load on health in paediatric populations.
3. Scientific evidence on the development of metabolism & changes in dietary needs and patterns from infancy to adolescence

**Fig. 2:**
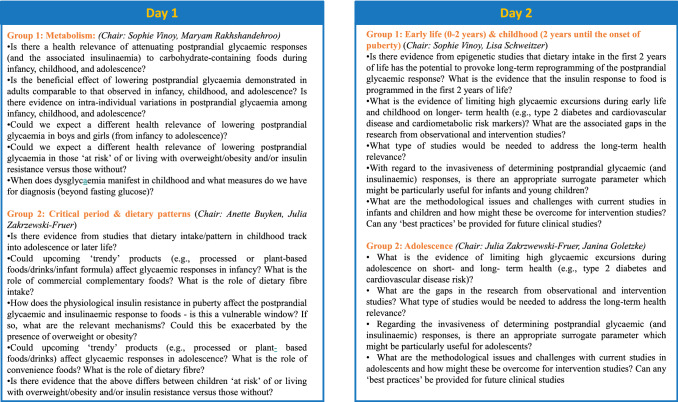
Questions for the breakout groups sent to all participants before the workshop

While the invited speakers gave overviews on the current state of evidence for selected topics (see ‘summary of the talks’ section), the leading objective of the breakout group discussions was to gather and discuss additional knowledge, potentially not addressed during the talks, and to identify research gaps regarding the effects of limiting glycaemia and insulinaemia on short- and longer-term health in paediatric populations. During the workshop preparation, the organising committee (Supp Table 1) compiled a list of questions (see Fig. 2) which were discussed during each breakout session. In total, four breakout groups were organized, with each being assigned specific topics for discussion. The first two breakout group discussions (day 1) addressed the first workshop session by discussing questions related to (1) metabolism, and (2) critical periods and dietary patterns, separately for infancy, childhood and adolescence, where applicable. The two breakout group discussions on day 2 addressed the second and third workshop session on mechanistic evidence and learnings from observational and intervention studies with one group focusing on (3) infancy and childhood and the other on (4) adolescence. The aims in each breakout group discussion were the following:

- To summarize and critically reflect on the presented and any additional knowledge.
- To specify methodological challenges.
- To identify gaps in research.
- To compile the need for future studies addressing these challenges.

Where applicable and/or possible, the discussions distinguished between the following three paediatric periods:

- Infancy, i.e., the first two years of life.
- Childhood, i.e., 2 years until pubertal onset.
- Adolescence, i.e., pubertal onset until the end of puberty (notwithstanding that the definition and methods used to assess puberty vary between studies, e.g., chronological age used as an indicator, pubertal markers assessed directly).

Participants were asked to select their preference for one of the two breakout sessions offered on each day (see Fig. 2) and were provided with the list of questions to be addressed in each of these breakout group discussions before the workshop. Each breakout group discussion was chaired by two members of the Organising Committee who provided summaries of the main outcomes at the end of the workshop. Of the total number of persons participating in the workshop on day 1 (54) and day 2 (49). For the breakout sessions, participants were allowed to choose the rooms and they were equally distributed in each breakout room. Based on the summaries from all four breakout group discussions, key themes were extracted (see ‘summary of breakout groups’ discussions) and subsequently summarized (see box 3).

## Results

The results consist of both the summaries of the invited talks and the summary from the breakout group discussions.

## Summary of talks

### Evert van Schothorst: early-life and nutritional programming in mice: role of carbohydrates

The process of nutritional programming by which nutrition in early life during pregnancy, lactation, weaning and early post-weaning, exerts permanent effects upon the developing foetus, providing windows of opportunities to combat metabolic diseases in adulthood [16, 17]. One of the three macronutrients, carbohydrates, might also offer such opportunities, as has been shown recently for proteins and fats [16].

Investigating the nutritional effects in an in vivo situation, a mouse model for human diseases, we previously showed that solely the change in starch composition (not its quantity), in a high fat diet background given to adult wildtype male mice, resulted in impressive beneficial health effects in response to the low digested or low GI starch type compared to the highly digestible or high GI starch type [18]. In this case, a 100% amylopectin starch was used as highly digestible, and a mix of 40% amylopectin and 60% amylose as low digestible starch. With similar food and energy intake over 14 weeks, the low GI starch type resulted in lower body weight, lower adiposity, improved glucose tolerance and improved insulin sensitivity in muscle and adipose tissue [18].

A lower digestibility might also result in increased fermentation by gut microbiota, a phenomenon gaining attraction due to, for example, the appearance of short chain fatty acids (SCFAs) and their beneficial health effects besides other effects the intestinal microbiota can have on the host´s health. One of those effects is the reduced availability of dietary energy, simply because the fermentation gases released, like methane or hydrogen, are exhaled and so energy is expended. Using an indirect calorimetry system, which measures inhaled and exhaled air of mice in their cage, equipped with supplemental fermentation gas sensors for hydrogen and methane, the low GI versus high GI diet yielded significantly higher hydrogen levels as quickly as within 12 h after first exposure to the diets [19]. Upon intervention in young post-weaning mice from three to six weeks of age, both males and females showed the same circadian rhythm of hydrogen release with higher levels in dark phase compared to light phase, as mice are nocturnal animals. Indeed, the low GI diet also led to increased levels of intestinal SCFAs [19]. However, when this period was followed by an obesogenic high fat diet for nine weeks, nutritional programming effects on physiological parameters were lacking [20].

Comparing the monosaccharides glucose, fructose and galactose showed differential effects for galactose, but not fructose. In detail, when post-weaning mice were fed an isocaloric diet for three weeks containing half of the carbohydrate fraction as starch and the other half as only glucose, versus fructose (both at 32en%) no differential direct or nutritional programming effects were seen in physiological and metabolic parameters [21]. In contrast, when half of the glucose was replaced by galactose, thus mimicking extended lactose intake following weaning for three weeks, this resulted in clear nutritional programming effects after the nine weeks obesogenic diet intervention; yet, in females only, decreased body weight, fat mass, lower serum leptin and insulin levels, and a reduced insulin resistance by extended early-life galactose intake were observed [22]. In adipose tissue, lower expression of, for example, insulin signalling markers were seen. Finally, repetition of the study design to focus also on the direct effects of galactose after the three weeks intervention showed a significantly decreased inflammatory state, which was confirmed by circulatory protein levels [23].

In conclusion, carbohydrates, and specifically starch type (available and resistant fractions) can have profound effects on digestibility and, as a result, microbiota activity, while the type of monosaccharides included in the early-life post-weaning diet can have direct as well as nutritional programming effects when galactose partly replaces glucose.

### Antonio ceriello: mechanistic insights—focus on human studies

Over the past few decades, postprandial GR has emerged as an important target for diabetes management since well-established evidence demonstrates that better glycaemic control is obtained by targeting also postprandial GR in addition to fasting plasma glucose [24, 25]. Also, evidence shows that high postprandial GR might be an independent risk factor for cardiovascular complications [24, 25].

Common practice procedures suggest targeting postprandial GR at 2 h after the start of meals. However, both the American Diabetes Association (ADA) and the International Diabetes Federation (IDF) guidelines also suggest targeting PPG after 1 h from the start of meals [25]. It is very important to understand why such a short-term increase (1 h) of plasma glucose might have a deleterious effect on health [26]. Endothelial dysfunction is an early abnormality, which can predispose to future cardiovascular events. In vitro studies, as well as studies in animals and in humans show that high glucose levels at 1 h postprandial are sufficient to induce endothelial dysfunction [27–33]. Oxidative stress produced by acute hyperglycaemia generates endothelial dysfunction, which, in turn, leads to CVD [27–34]. Moreover, oxidative stress is currently considered the key pathogenic mechanism leading to diabetic complications. A recent study supports the possibility that exposure to high glucose levels at 1 h is a sufficient stimulus for inducing oxidative stress in endothelial cells and animals [33]. Short term exposure to high glucose activates a stable left shift of the glucose concentration and reactive oxygen species production at mitochondrial level [33]. Interestingly, this phenomenon has a dose–response curve. The key question that remains, however, is why hyperglycaemia at 1 h should be considered more deleterious than that at 2 h. The simple explanation is that, at 1 h, the level of glycaemia reached is generally higher than that at 2 h, both during OGTT and meals. In T2D, the post breakfast peak value is observed between the first and second hour (approximately at 70 min) after the start of a meal irrespective of the degree of glycaemic control assessed by the HbA1c level, while the 2-h post breakfast glucose value is already within the decreasing slope. In other words, the problem seems to be not the timing, but rather the higher level of glycaemia, which is usually reached at around 1 h.

- In any category of glucose tolerance, endothelial dysfunction has been shown to be directly correlated to the level of glycaemia reached during OGTT [30];
- ‘‘Glucose spikes’’, more than any other value of glycaemia, are a stronger predictor of carotid intima media thickness;
- Endothelial dysfunction is also directly correlated with the ‘‘glucose spikes’’ [31];
- Endothelial function is always worse at 1 h postprandial when compared to 2 h postprandial, both during OGTT and meals, which is in accordance with the higher level of glycaemia observed at that time [30];
- Hyperglycaemia decreases myocardial function, with this effect being related to the level of glycaemia reached [28];
- Other risk factors for cardiovascular diseases, such as inflammation and thrombosis activation, follow the same pattern of endothelial dysfunction as during acute hyperglycaemia [32];
- Oxidative stress, which induces endothelial dysfunction, is also generated by acute hyperglycaemia and is higher at higher levels of glycaemia [30].

Finally, recent evidence stresses the role of hyperglycaemia itself, by favouring beta-cell functional damage, suggesting that the higher the glucose level, the more damaging is its effect on beta cells [35]. These findings may help explain why 1 h glycaemia during OGTT is more predictive of the development of T2D than values taken at 2 h [35]. All the available human studies were performed in adults; however, considering the mechanisms reported above, there is no doubt that PPG can be similarly dangerous in children and adolescents. Therefore, it is worth recommending PPG control at any age. Moreover, even with non-existing specific evidence in children and adolescents, the importance of the 1 h timepoint should be also considered in paediatric populations. Finally, recent evidence stresses the role of hyperglycaemia itself, by favouring beta-cell functional damage, suggesting that the higher the glucose level, the more damaging is its effect on beta cells [35]. These findings may help explain why 1 h glycemia during OGTT is more predictive of the development of T2D than values taken at 2 h [35]. All the available human studies were performed in adults; however, considering the mechanisms reported above, there is no doubt that PPG can be similarly dangerous in children and adolescents. Therefore, it is worth recommending PPG control at any age. Moreover, even with non-existing specific evidence in children and adolescents, the importance of the 1 h timepoint should be also considered in paediatric populations.

### Antje Körner: detection of early glycaemic comorbidities in obesity

#### Childhood obesity is a chronic, relapsing and progressive disease

Even though leading professional societies acknowledge obesity to be considered as a disease during the paediatric period, it is still often misconceived as failure to adapt to a healthy lifestyle. The definition of obesity between the ages of 2 and 18 years is not based on absolute numeric cut-offs in body mass index (BMI), as in adults, because the dynamics of child development need to be taken into account. Usually, it is defined by the highest percentiles of the general population distribution of BMI. In Germany, obesity is defined above the 97^th^ percentile for chronological age and sex of the national BMI distribution during childhood. Based on this number, in the last two decades, the prevalence and the degree of obesity in German children increased dramatically [36]. Based on longitudinal data, there is a critical period with the highest risk to develop obesity in childhood and adolescence, it is the so-called “vulnerable period” between 2 and 6 years of age with the highest weight gain. Once obesity has manifested, the likelihood of persistence into adulthood is more than 90% [37].

The comorbidities of childhood obesity are frequently diagnosed during adulthood with dramatic consequences. Indeed, it is associated with a shortened life expectancy compared to populations without obesity. In the Pima Indian community, it has been clearly shown that childhood obesity increased the death’s number before 40 years by 10% compared to subjects without obesity [38]. This long-term health deterioration linked to obesity comorbidities is already detectable during childhood with the emergence of markers as [39–41]:

- Alteration in growth: the children with obesity are taller from 4 to 10 years and then, their growth is slower during puberty [39].
- Cardiovascular dysfunction: the alteration of the endothelial function and cardiac remodelling [41].
- Metabolic complication: development of hepatic steatosis which is involved in dysglycaemia [42].

#### The metabolic side of comorbidity emerging from childhood obesity

The prevalence of diabetes is increased among those with childhood obesity especially for T2D [43]. This young onset of T2D is a phenotype associated with high mortality, more complications and unfavourable cardiovascular disease risk factors, even compared to type 1 diabetes. Yet, even in this very unfavourable health situation, there is a window of opportunity. For adolescents who are obese with a successful reduction of weight, there is a clear improvement and even remission of cardiometabolic risk [44], even better than compared to adults. Thus, there is a need to detect the risk of metabolic comorbidity early. One crucial step is to be able to characterize the metabolic status of the children. The current main criteria to classify (pre)diabetes are (American Diabetes Society):

- 2 h plasma glucose in OGTT (oral glucose tolerance test).
- Fasting glucose.
- HbA1c.

Based on these official criteria, only 12.6% of children with obesity are diagnosed with impaired glucose metabolism. However, the correlation of glucose-based parameters with BMI SDS is poor.

Even if these criteria have been used for decades, they do not entirely reflect the progressing hyperinsulinemia in children with obesity as the first sign of insulin resistance. We have to take into account the modulation of insulin sensitivity during growth. This parameter is strongly deteriorated with the increase in BMI of children. If we consider the in-depth insulin response recorded during an OGTT, the insulin response is doubled in children with obesity to the same amount of glucose when compared to normal weight children. Thus, there is a need to add insulin secretion as a criterion to determine metabolic dysfunction. The surrogate markers which include more than fasting blood glucose or insulin only, are better correlated with insulin sensitivity, especially with the OGTT derived indices. For example, the IS-Matsuda Index as an index of insulin sensitivity is already impaired in more than half of 6-year-old children with obesity, which shows that potential metabolic dysfunction can occur very early in life. Based on the IS-Mastuda Index, the new calculation of the percentage of children with obesity and insulin resistance was much higher than the 12.6% identified with the three official criteria. Indeed, around 50% or more of these children had insulin resistance. Also, these OGTT derived indexes are much more efficient to discriminate children in the prediction of future dysglycaemia, i.e., impaired glucose tolerance, impaired fasting glucose and finally development of type 2 diabetes. To conclude:

- Obesity is a chronic disease which emerges in childhood. Once manifested, it has a high likelihood to persist.
- Childhood obesity causes comorbidity characterised by early progressive dysglycaemia, which emerges even in childhood.
- There is an urgent need to better diagnose with predictive markers to identify children with a high risk of progressive cardiometabolic deterioration.

### Anette Buyken: relevance of the dietary glycaemic index and glycaemic load for health in childhood and adolescence—insights from observational and interventional studies

Data from adults suggest a major role of the dietary GI and GL both in weight maintenance [45] and the prevention [7] and treatment of T2D [46]. A potential relevance of the dietary GI and GL for health in childhood and adolescence is underpinned by the fact that high GI foods make a major contribution to the diet of children and adolescents [47, 48] and that their relevance increases with age [49]. Children and adolescents from Asian populations may generally be at higher risk of higher dietary GI values: mean dietary GI in Japanese were GI = 63 at ages 1–6 and GI = 67 at ages 15–19 [47], i.e., notably above values observed in European and Australian paediatric populations [49, 50].

In addition, recent data suggest that high GI infant formulas and high GI weaning foods do exist, yet only few data are available on the extent of the problem. The new GI table contains merely 13 values for infant formulas and 11 values for follow-on formulas and 29 values for complementary/weaning foods measured according to ISO standards [1]; for several commonly consumed weaning foods (e.g., vegetable-meat purees) the data are lacking. Human milk has been shown to have a GI and insulinaemic index comparable to that of a typical lactose-based formula; hence, human milk is unlikely to have “programming effects” due to differences in postprandial glucose homeostasis [51]. Taken together, data presently available do not allow an estimation of dietary GI or GL in infancy. In addition, it may be problematic to extrapolate GI values currently measured among adults to glycaemic responses of infants: a study measuring the capillary glucose value at 60 min postprandial in four-eight months old infants found that glucose response to a follow-on formula with isomaltulose (i.e., lower GI) was higher than to a conventional formula after a 4-week period of consumption [52]. However, since only one measurement was possible in this study on infants, it is possible that the measurement was too early or too late to capture potential benefits of an isomaltulose formula.

Among adolescents, glycaemic response to a high GI breakfast was significantly higher than to a low GI, yet that same study showed a clearly higher insulinaemic response among girls compared to boys, presumably attributable to the more advanced stage of puberty among girls [53]. While the ranking of high versus low GI meals was retained, puberty may resemble another critical period for the regular consumption of high GI foods, since a compensatory insulin response is needed to overcome pubertal insulin resistance [54]. This may in fact be exacerbated by the presence of overweight, as illustrated by a higher glycaemic response to a high GI breakfast in comparison to a low GI breakfast among overweight adolescent girls but not among non-overweight adolescent girls [55].

Finally, a preferred consumption of carbohydrates from lower GI sources is associated with benefits for overall nutrient adequacy [56]. Of note, a higher dietary GI is associated with higher starch intake and a lower overall sugar intake, yet not or only weakly associated with dietary fibre in different age groups (2–16 years) [49]. Hence, dietary advice for lowering dietary GI/GL should be specifically tailored to top contributors to dietary GL, which vary by ethnicity and age.

Interventional evidence from the DIOGENES study conducted among 827 children aged 5–18 years reported a significantly larger reduction in the percentage of overweight or obesity in the group consuming a high-protein low GI-diet, yet no isolated effects of GI or protein [57]. A meta-analysis of nine intervention studies among children and adolescents found no significant effects of low GI diets on a range of anthropometric measures when compared to high GI diets, yet the pooled estimate from the four studies assessing insulin resistance (HOMA-IR), suggested a superiority of low GI/GL diets [13]. However, the PREVIEW study on 126 adolescents with overweight and increased insulin resistance did not confirm a difference in measures of insulin resistance between a medium protein/higher GI diet (target 15% protein, GI >  = 56) and a high protein/lower GI diet (target: 25% protein, GI <  = 50) after one or two years [58]. This study reported substantial problems with the feasibility of the diets, compliance was low and dropout very high (only 49 adolescents completed the 2 year follow-up). Hence, notwithstanding the potential benefits of a diet lower in dietary GI, long-term implication may require additional support, e.g., by changing the nutritional environment offered to adolescents in school and other eating-out-of-home sectors (Public Health England latest report on Sugar reduction 2020). The few studies investigating the prospective relevance of dietary GI/GL on metabolic outcomes do not support an important role of dietary GI/GL for health outcomes either in childhood [59, 60] or adolescence [61–66]. Only two studies examined the prospective relevance of dietary intake in adolescence for adult health outcomes [50, 65], with data from Portugal suggesting that dietary GI/GL in adolescence tracks into adulthood [65]. Data from the DONALD study, which estimated the dietary GI/GL from an average of five 3 day dietary records collected during adolescent years, suggest that a higher dietary GI during adolescence may be detrimental for insulin resistance, hepatic steatosis markers and markers of subclinical inflammation [50, 67] in young adulthood. Additional analyses revealed that these associations are restricted to evening intakes, i.e., that time of consumption during the day may be of long-term relevance for type 2 diabetes prevention [68]. These findings require replication in other long-term observational and/or interventional studies.

### Ute Alexy: development of dietary habits during infancy, childhood and adolescence

Food intake in children is mainly determined by preferences and aversions. In humans, the preference for the sweet taste and the aversion to sour and especially bitter tastes are innate. However, inherited preferences and aversions of tastes as well as likes and dislikes of flavours can be modified by exposure [69, 70], possibly through epigenetic programming [71]. This experience starts before birth, as flavours from the mothers’ diet are transmitted to the amniotic fluid. Postnatally, due to its high lactose content, breast milk tastes slightly sweet. Additionally, flavours in breastmilk reflect the foods, spices or beverages ingested by the mothers. Breast milk therefore tastes slightly different every day [69]. In fact, breast fed children are more willing to accept complementary food, are less picky, and fruit and vegetable consumption in childhood is higher than among formula fed counterparts [69].

During complementary feeding, it is recommended to offer a broad range of flavours by changing the types of vegetables or fruits and not adding any sugar, other sweeteners, or salt [70]. However, labelled total sugar content, from natural and added sources, of commercial complementary food products is generally high due to the widespread use of pureed fruits or juice [72]. As commercial complementary foods are commonly used in families [73, 74], the sugar content of commercial complementary food should be more regulated [72].

During childhood, sugar intake is high, although at least in Germany, free sugar intake decreases with age after a maximum around nine to ten years of age [75]. This is probably due to a decrease in sweet preference from early adolescence until adulthood [76, 77]. However, reports indicate that free sugar intake exceeded the 10% of energy intake limit [78] in all age groups [75]. This high sugar intake is accompanied by a low fibre intake in childhood and adolescence, which decreases with age [79, 80]. Adolescents’ diets are characterized by snacking moments [81, 82], a high consumption of fast food (i.e., convenience food purchased in self-service or carry-out eating places [83] or ultra-processed food, i.e., foods made from processed substances extracted or refined from whole foods, with little or no whole foods [84–86], and a trend towards plant-based foods [87]. As there is no standard definition of meals or snacks, the results of potential associations of snacking and diet quality with body weight status are mixed [81, 82]. Fast food and ultra-processed food have a high content of energy, sugar, fat, saturated fatty acids, and sodium in common, and may thus increase the risk of being overweight and contribute to a lower overall dietary quality [85, 86]. Children and adolescents on plant-based vegan diets had lower intakes of protein and dietary sugars, but a higher intake of carbohydrates and dietary fibre in particular when compared with omnivores. Vegetarian diets are in the middle range [87–89]. Food consumption data showed a more favourable food choice with a higher intake of legumes, nuts, and whole grain of children on a vegetarian and, in particular, a vegan diet compared with omnivores [90]. However, the long-term effects of adherence to these plant-based diets during childhood and adolescence are not yet clear. Data from adults indicate beneficial health outcomes and reduced carbon footprints among vegetarians and vegans [91], but a reduced bone mineral density and in vegans, an increased risk of fracture [92].

In general, the individual life periods should not be considered independently of each other, since dietary habits seem to be stable when children age and track at least weakly to moderately from childhood to adolescence and young adulthood [93, 94]. In conclusion, there is potential for improvement in the diets of children and adolescents (too much sugar, too little fibre, unfavourable food patterns in adolescence). However, dietary habits can be shaped—preferably from the beginning—through exposure. For this, a healthy nutrition environment is important.

## Summary of group discussions

The following sections summarize the discussions from all four breakout groups by key extracted themes. Overall, the discussion drew on (i) the questions sent to the participants (see Fig. 2), (ii) the contents of the talks from the experts (see chapters above) and (iii) additional input based on the expertise of the participants, as many of them were researchers in the field.

### Metabolism and mechanisms

Data on the metabolic responses to dietary manipulations reducing GR in infants are very limited. The group conceded that the extrapolation of data stemming from pre-pubertal children or adults to infants is often used as the ‘best available’ option in spite of known age-related metabolic differences and the limited evidence base available for pre-pubertal children. Nevertheless, the group agreed that learnings from adult studies suggest an important health relevance of glycaemia and insulinaemia also for paediatric populations. The group acknowledged that the shape of glucose and insulin response, the magnitude of response and the duration of the elevated response before returning to baseline all require further investigation in paediatric populations in a health context. Also, the frequency of glycaemic challenges should be considered and could play an important role for cardiometabolic risk factors.

The group agreed that gaps in research addressing adolescents include a more detailed assessment of both hormonal and behavioural changes, which are in turn related to postprandial glycaemia: In light of the physiologically occurring insulin resistance in puberty, where insulin sensitivity decreases during mid-puberty and recovers to pre-pubertal levels at the end of puberty, data on postprandial GR and IR to foods considering sex-specific pubertal stages is warranted. The interplay of postprandial glycaemia and insulinaemia with pubertal insulin resistance should be examined in the context of overweight/obesity and vice versa. Furthermore, measured GI data on local foods consumed in different ethnic adolescent populations are needed, acknowledging adolescence as a time period when own food choices are made that differ from family eating patterns.

Data on factors influencing postprandial glycaemic and insulinaemic responses in infants, children and adolescents are at best emerging. In pre-clinical studies, decreases in GI are associated with an increase in intestinal fermentation of carbohydrates leading to SCFA and H_2_ production, hence it cannot be disentangled whether benefits are attributable to the GI per se or the shift in fermentation. Moreover, clear sex-differences have been observed in a nutritional programming model in young and adult mice using different dietary monosaccharides. Some workshop participants considered that, due to the lack of data, overweight/obesity could serve as a proxy indicator of poor glycaemic control in infancy and childhood. Yet, others pointed out that inter-individual variations in GR and IR have been observed even among individuals with the same BMI. As in all age groups, when addressing glycaemia there is a need to consider the balance of dietary carbohydrate intake versus carbohydrate use, with physical activity being the most modifiable component of carbohydrate oxidation through the additional energy expenditure.

Generally, it was indicated that, as for other periods in life, focusing solely on glycaemia is likely not sufficient for cardiovascular disease prevention, as oxidative, inflammatory and other metabolic processes as well as interactions between macronutrients also require consideration. In addition, several confounding factors (diet-related or diet-unrelated) need to be accounted for. Finally, the workshop participants emphasized that a valid and precise diet characterization as well as defining appropriate intervention and control diets are cornerstones for future studies.

### Learnings from observational and intervention studies

It was highlighted that research in infants, children and adolescents is associated with several methodological challenges and ethical concerns which, in turn, have led to the limited evidence. Minimally invasive methods as alternatives for invasive blood drawing and suitable surrogates for glucose metabolism are warranted particularly for infants and children (e.g., continuous glucose monitoring, biomarkers in saliva, stable isotopes in the breath of breastfed infants). Moreover, the possibility to modulate the diet in studies with infants and children is limited, yet necessary since many dietary constituents interfere with glucose metabolism. It was proposed that studies involving infants could, for example, take advantage of everyday-life formulas chosen by parents that differ in their predominant carbohydrates (e.g., lower-GI vs. higher-GI carbohydrates), thus minimizing the need for active dietary manipulation. For studies involving adolescents, improving compliance seems pivotal and more data on how to do this is urgently needed. Studies in adolescents might benefit from the use of new technologies of (flash) glucose monitoring (FreeStyle Libre, Dexcom, etc.). The workshop participants reported on validation studies in adolescents and university students showing plausible results for patterns and fluctuations, but not for actual values. Study compliance was reported as promising, indicating that this technology might be a useful surrogate marker of postprandial glycaemia.

Beyond these issues concerning short-term GR and IR, there was consensus that data linking postprandial responses to longer-term health outcomes in interventional and observational studies is required. According to the participants, evidence from epigenetic studies in support of a long-term reprogramming of the postprandial GR and IR due to dietary intake in infancy is very limited. To date, epigenetic and clinical studies focus on programming effects of diet during pregnancy. Even though some studies indicate a benefit of low-GI diets consumed during pregnancy on body weight of the offspring, it was pointed out that the overall evidence is rather inconclusive. To understand the potential programming of lowering postprandial glycaemia in infants and children, the group discussed that one could also perhaps learn, among others, from the early protein hypothesis (which postulates that higher protein intakes in the first months of life increase the risk of subsequent obesity possibly by inducing distinct hormonal responses (e.g., stimulating insulin or IGF-1 secretion)).

With respect to studies starting in adolescence, it was discussed that low-GI diets might help to preserve insulin capacity in the long-term. The group felt that valid predictors such as cardiometabolic markers examined in observational studies might serve as proxies for longer-term health and hence partly overcome the problem of the long time-lag between cause and effect. Nonetheless, it was agreed that the long latency period between exposure and disease manifestation (e.g., T2D, cardiovascular disease) hampers conclusion on causality.

### Critical periods and dietary patterns

The group felt it was important to clarify whether the dietary GI or GL tracks from infancy to adolescence or even adulthood, as currently longer-term within-person data is lacking. There is general, but not necessarily GI-specific, evidence supporting a tracking of dietary intake and dietary pattern from infancy, childhood and adolescence to later life, with associations between dietary intake/pattern becoming stronger when the age gap is narrower (e.g., adolescence to adulthood versus childhood to adulthood). Yet, the available research is generally limited by methodological difficulties in assessing habitual diet and the common assessment of tracking at a limited number of time points (commonly only two, i.e., baseline and endpoint). Furthermore, differences in dietary assessment methods across the literature limit inter-study comparisons. It was acknowledged that it is important to differentiate between the individual and the environment; for example, the family and home environment govern dietary intakes in younger children, whereas individual choices as well as peer groups, school environment or other eating-out-of-home sectors become more important in adolescence. Unfortunately, there is a lack of data on the effect of these changes in dietary habits on metabolism.

Factors beyond physiology were also discussed with respect to the vulnerable window of adolescence. In particular, lifestyle factors such as physical activity, which are linked to insulin resistance, might be of special relevance, since physical activity levels commonly decline from childhood to adolescence, particularly among girls. In addition to carbohydrate quantity and quality, meal timing in adolescence should be considered. Specific concerns expressed for this population included breakfast skipping and high intakes of higher GI carbohydrates in the later evening, which may interact with individual chronotypes. It was also stressed that among some adolescents (changes in) food choices may entail high levels of fructose intakes, a specific concern for adolescents who are overweight/obese due to the risk of developing non-alcoholic fatty liver disease.

Finally, the group discussed whether adolescents may be more vulnerable to the impact of food processing on the GR to foods when compared to infants and children. One of the obstacles in clarifying this is the fact that the definition of processed foods is currently not standardized including the effect of food processing on GR. In addition, concern was raised that the focus of public health initiatives on sugar reduction in convenience foods may carry a risk that ‘sugar’ could be replaced with processed high GI carbohydrates, which would entail a greater glycaemic response than the standard product. Specific food processes (e.g., heat treatment) could change the quality of both proteins and carbohydrates by modifying their structure, resulting in different digestion and absorption dynamics and different postprandial responses. Furthermore, generally, the European and U.S public are not well-informed on the meaning of the ‘GI’; thus, increasing this knowledge in younger generations i.e., in educational settings, seems important.

## Conclusion

**Fig. 3:**
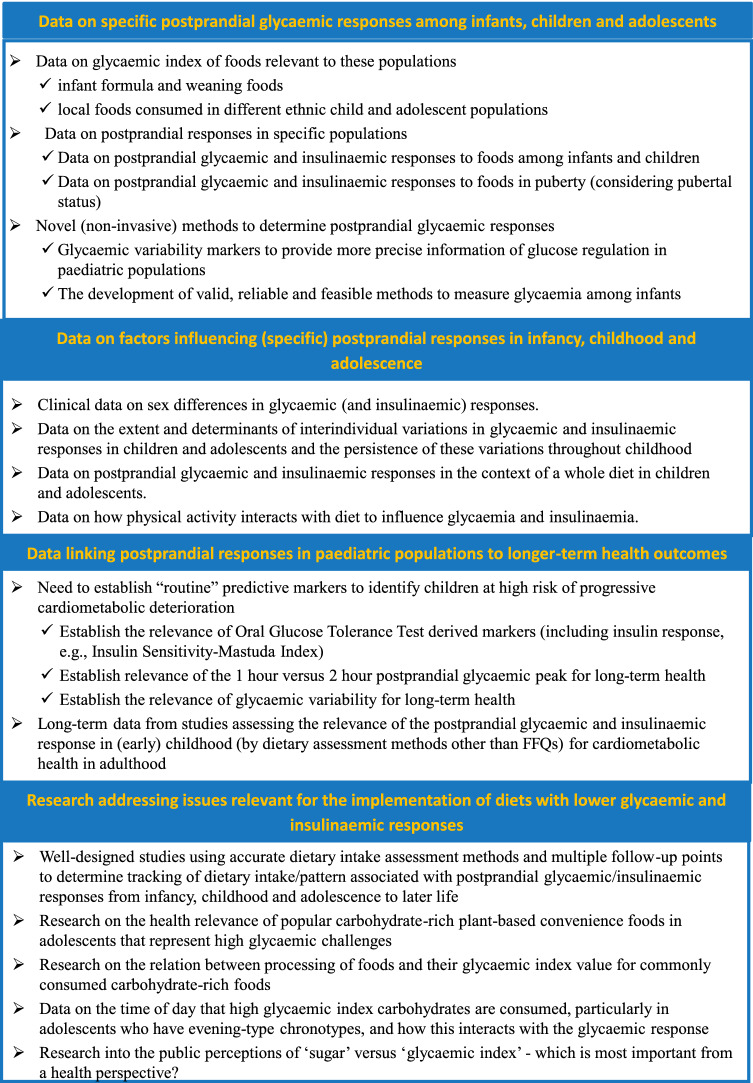
Gaps in research identified during the course of the workshop—points emerged from both invited talks and the breakout group discussions

In summary, based on the shared consolidated scientific knowledge and recent advances, the workshop led to the consensus on the crucial role on health of postprandial GR in paediatric population, even though a lack of scientific data has been identified regarding detailed glucose and insulin profiles in response to foods commonly consumed by this population, as well as a lack of long-term evidence. Furthermore, the major gaps (see Fig. 3 for details) identified by the expert talks and the subsequent group discussions were (i) the lack of detailed glucose and insulin profiles in response to foods commonly consumed among infants, children and adolescents as well as data on postprandial responses in these paediatric populations, (ii) appropriate specific predictors in childhood and adolescence for long-term health on the other hand and (iii) long-term follow-up from intervention studies and/or prospective observational studies elucidating the relevance of postprandial glycaemic and/or insulinaemic excursions for long-term health (i.e., in adulthood).

## Supplementary Information

Below is the link to the electronic supplementary material.

Supplementary file1 (PDF 128 KB)
