# Supplementary material for: Health relevance of lowering postprandial glycaemia in the paediatric population through diet’: results from a multistakeholder workshop
Source: Eur J Nutr. 2022 Dec 19;62(3):1093–107. doi: 10.1007/s00394-022-03047-y (PMC10030539; doi:10.1007/s00394-022-03047-y)
Supplement: Supplementary file 1 — Supplementary file1 (PDF 128 KB) [file 394_2022_3047_MOESM1_ESM.pdf]

A: Participants during Day 1

Participants	Affiliation
Adrienne Weiss	Yili Innovation Center Europe
Alena Schadow	Universität Paderborn
Andreas Pfeiffer	Charité Universitätsmedizin Berlin
Anette Buyken[**,&]	Universität Paderborn
Antje Körner[&]	University of Leipzig
Aurelie Delanoue	Danone Nutricia Research
Bettina Krueger	Universität Paderborn
Bianca Stutz	Universität Paderborn
Carolien van Loo-Bouwman	Yili Innovation Center Europe
Cecile Singh-Povel	FrieslandCampina
Claudio Maffeis	University of Verona
Delphine Saulnier	Novozymes
Elaine Vaughan	Sensus
Elin Östman	InnovaFood AB
Emilie Martinet	Mondelez
Evert van Schothorst[&]	Wageningen University
Harm van Baar	Knowledge center sugar & nutrition
Ilaria Calabrese	University of Naples Federico II
Ingrid Revheim	University of Bergen
Janina Goletzke[#]	Universität Paderborn
Javier Martín-Tereso	Trouw Nutrition
Jimmy Chun Yu Louie	The University of Hong Kong
Jolie Dubbers-van Harten	Danone Nutricia Research
Jose Maria Lopez[#]	Abbott Nutrition
Jose Villa Risriguez	Diana Nova
Julia Fruer[#]	University of Bedfordshire
Kathrin Jansen	Universitäts kinder klinik Bochum
Laura Barrett	Loughborough University
Linette Pellis	Ausnutria B.V.
Maria Angela Guzzardi	National Research Council (Italy)
Marieke Abrahamse	Danone Nutricia Research
Maryam Rakhshandehroo[#]	Danone Nutricia Research
Mette Axelsen	EasyWise AB
Mirela Nedelescu	National Institute of Public Health
Nikoleta Stamataki	University of Manchester
Patricia Iozzo	National Research Council (Italy)
Petra Wendorf-Ams	Danone Nutricia Research
Rebecca Jones	University of Bedfordshire
Renaud Mestdagh	Cargill
Ricardo Rueda-Cabrera	Abbott Nutrition R&D
Sahar Afeef	Loughborough university
Saleha Rahman	Cuemath

Simon Bøge Riis	Nottingham Trent University
Sophie van Oppenraaij	Danone Nutricia Research
Sophie Vinoy*	Mondelez Int R&D
Stefan Kabisch	Charité
Stephan Theis	BENEO
Stewart Forsyth	University of Dundee
Therese Hjorth	Chalmers
Thomas Heidebach	Archer Daniels Midland
Ulrike Spielau	University of Leipzig
Ute Alexy[#]	University of Bonn
Vicenta Campayo	Cargill
Willie Woestenenk	Danone Nutricia Research

B: Participants during day 2

Participants	Affiliation
Adrienne Weiss	Yili Innovation Center Europe
Anette Buyken**[,&]	Universität Paderborn
Antonio Ceriello[&]	IRCCS Multimedica
Aurelie Delanoue	Danone Nutricia Research
Bettina Krueger	Universität Paderborn
Bianca Stutz	Universität Paderborn
Carolien van Loo-Bouwman	Yili Innovation Center Europe
Cecile Singh-Povel	FrieslandCampina
Claudio Maffeis	University of Verona
Delphine Saulnier	Novozymes
Elaine Vaughan	Sensus
Elin Östman	InnovaFood AB
Euridice Castaneda	H&H
Evert van Schothorst[&]	Wageningen University
Ilaria Calabrese	University of Naples Federico II
Ingrid Revheim	University of Bergen
Jaap Keijer	Wageningen University
Janina Goletzke[#]	Universität Paderborn
Janna van Diepen	Reckitt
Jimmy Chun Yu Louie	The University of Hong Kong
Jose Maria Lopez[#]	Abbott Nutrition
Julia Fruer[#]	University of Bedfordshire
Kathrin Jansen	Universitäts kinder klinik Bochum
Laura Barrett	Loughborough University
Linette Pellis	Ausnutria B.V.
Lisa Schweitzer[#]	BENEO
Maria Angela Guzzardi	National Research Council (Italy)
Marieke Abrahamse	Danone Nutricia Research

Maryam Rakhshandehroo[#]	Danone Nutricia Research
Mette Axelsen	EasyWise AB
Mirela Nedelescu	National Institute of Public Health
Nikoleta Stamataki	University of Manchester
Penbe Ecem Mısırlıoğlu	Mısırlıoğlu
Petra Wendorf-Ams	Danone Nutricia Research
Renaud Mestdagh	Cargill
Renske van Asten	Kenniscentrum suiker & voeding
Ricardo Rueda-Cabrera	Ricardo Rueda-Cabrera
Sahar Afeef	Sahar Afeef
Saleha Rahman	Cuemath
Shila Shafaeizadeh	Danone Nutricia Research
Sophie van Oppenraaij	Danone Nutricia Research
Sophie Vinoy[*]	Mondelez Int R&D
Stephan Theis	BENEO
Stewart Forsyth	University of Dundee
Therese Hjorth	Chalmers
Thomas Heidebach	Archer Daniels Midland
Ulrike Spielau	University of Leipzig
Vanda Cristóvão	SESARAM
Willie Woestenenk	Danone Nutricia Research

**: Chair of the Organising Committee

*: Vice-Chair of the Organising Committee

[#]: Organising Committee member

[&]: Speaker
